# Cranial Osteopathy: Obscurantism and Enlightenment

**DOI:** 10.7759/cureus.4730

**Published:** 2019-05-23

**Authors:** Bruno Bordoni, Bruno Morabito, Marta Simonelli

**Affiliations:** 1 Cardiology, Foundation Don Carlo Gnocchi, Milan, ITA; 2 Osteopathy, School of Osteopathic Centre for Research and Studies, Milan, ITA; 3 Osteopathy, French-Italian School of Osteopathy, Pisa, ITA

**Keywords:** osteopathic, fascia, quantum physics, neurophysiology, cranio

## Abstract

The application of cranial osteopathic manipulative medicine (OMM) is always controversial in the literature. Primary respiration related to the movement of spheno-basilar synchondrosis in the adult goes against the knowledge of complete ossification that occurs at this articulation after the pubertal phase. The idea that the operator's hands can communicate with the meninges is difficult to accept. The anatomy shows us that the fascial system involves the meninges and that from the microcellular point of view there are no layers that divide one tissue from another. The backing of new sciences, such as quantum physics, suggest that cranial palpation allows the osteopath to come into contact with the meninges. Recent scientific evidence shows that meningeal afferents can affect extracranial areas and that the pericranial musculature itself is able to influence these afferents. The article highlights some reflections in support of cranial osteopathy, based on scientific information that could help the osteopath to improve clinical work.

## Introduction and background

In the scientific landscape, the approach with the cranial osteopathic manipulative medicine (OMM) is much debated [1]. At present, there is still no absolute recognition of the effect of manual cranial manipulations in the field of international literature. The latest revisions on the OMM are negative, describing the fallacious scientific depth and the poor methodology applied in carrying out the research, thus relegating this medical discipline to the borders of the credible [2-3]. We know that the synchondrosis between the occipital bone and the sphenoid bone, articulation underlying the concept of primary respiration and cranial bone movement, begins to ossify from 11-13 years to complete ossification at the end of puberty [4]. The process begins at the level of the endocranial surface, to finally continue ectocranially [4]. From this point of view, the cranial model devised by Dr Sutherland should be reviewed. Brain meninges are subject to physiological calcification, with age or due to previous head trauma or craniocervical surgery [5-7]. The ossification puts into question the manual techniques for draining the dura mater's sinus or for influencing the cerebrospinal fluid (CSF) or the lymph of the glymphatic system [8-10]. The choroid plexus region can ossify bilaterally [5]. This information reminds us that the production, circulation and absorption of liquor is still a matter of debate by researchers [11]. Probably, liquor does not circulate, but disperses depending on the molecular weight of its components, and is absorbed differently by the ventricles and sub-arachnoid space [11]. Scholars who are not used to putting their hands on the patient's skull are inclined to rely only on this information, in order to demonize the OMM, but every coin has always two sides. To understand a phenomenon one must not be prejudicial, but follow every information and scientific field to correctly define the contours of the event to be studied. If such a research strategy is lacking, full awareness cannot be reached (knowing or not being able to know), but a self-imposed scientific limit is reached, which does not correspond to the final goal of the scientist and scholar. In this way, obscurantism is created.

## Review

**Figure 1:**
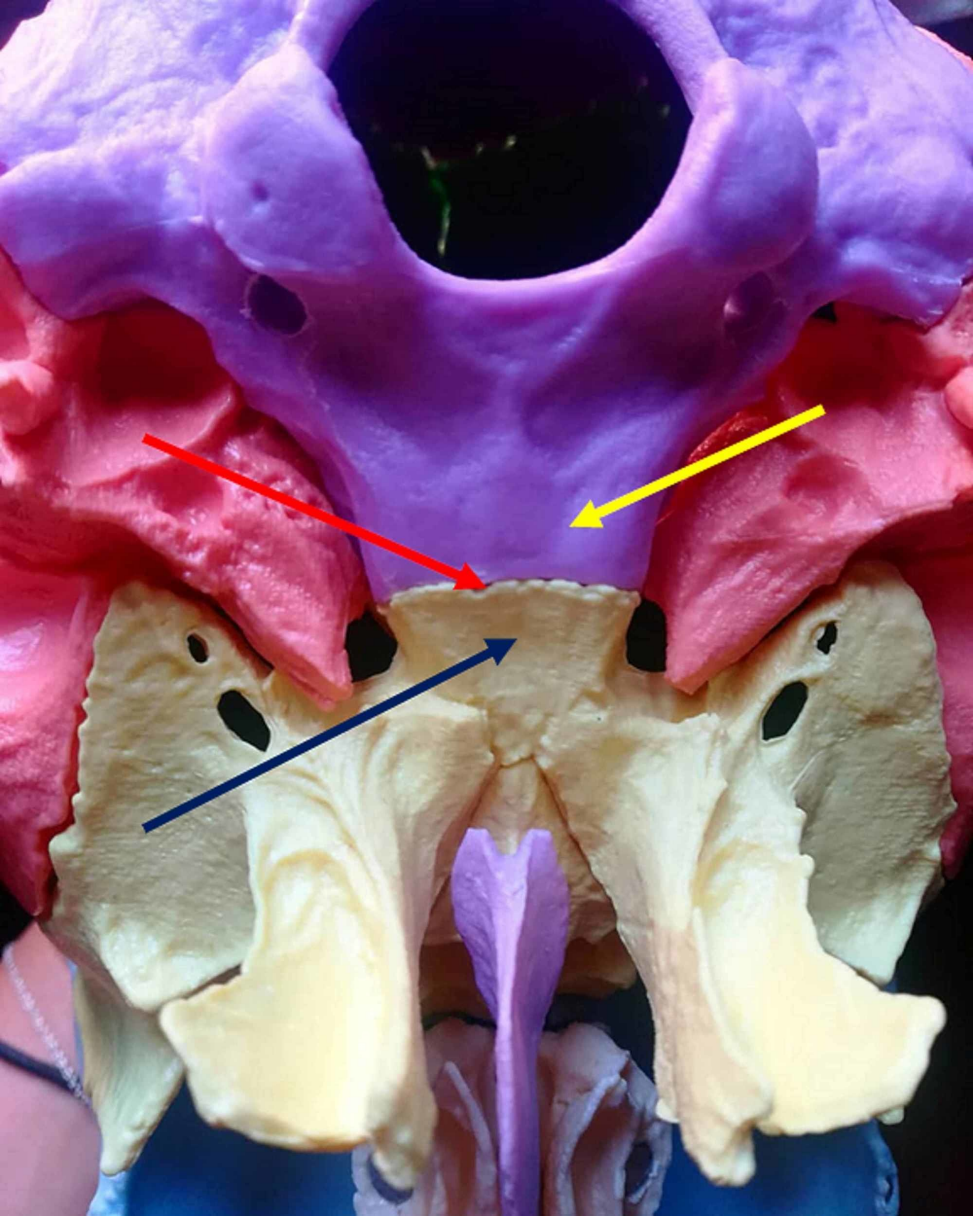
The figure illustrates the synchondrosis articulation between the occipital bone and the sphenoid bone, with a view of the base of the skull. The red arrow indicates the synchondrosis articulation; the yellow arrow indicates the basiocciput; the blue arrow indicates the body of the sphenoid.

Metanalytic reviews forget which are the foundations of evidence-based medicine, that is, the fusion of the operator's clinical experience, the patient's experience and experimental research: "External clinical evidence can inform, but can never replace, individual clinical expertise, and it is this expertise that decides whether external evidence applies to individual patients at all, if, how it should be integrated into a clinical decision" [12]. The synchondrosis between the occipital bone and the sphenoid bone when ossified does not create movement (flexion-extension), but the most recent scientific notions show that most cranial sutures or synarthrosis are patent, even in very old subjects (Figure 1) [4].

**Figure 2:**
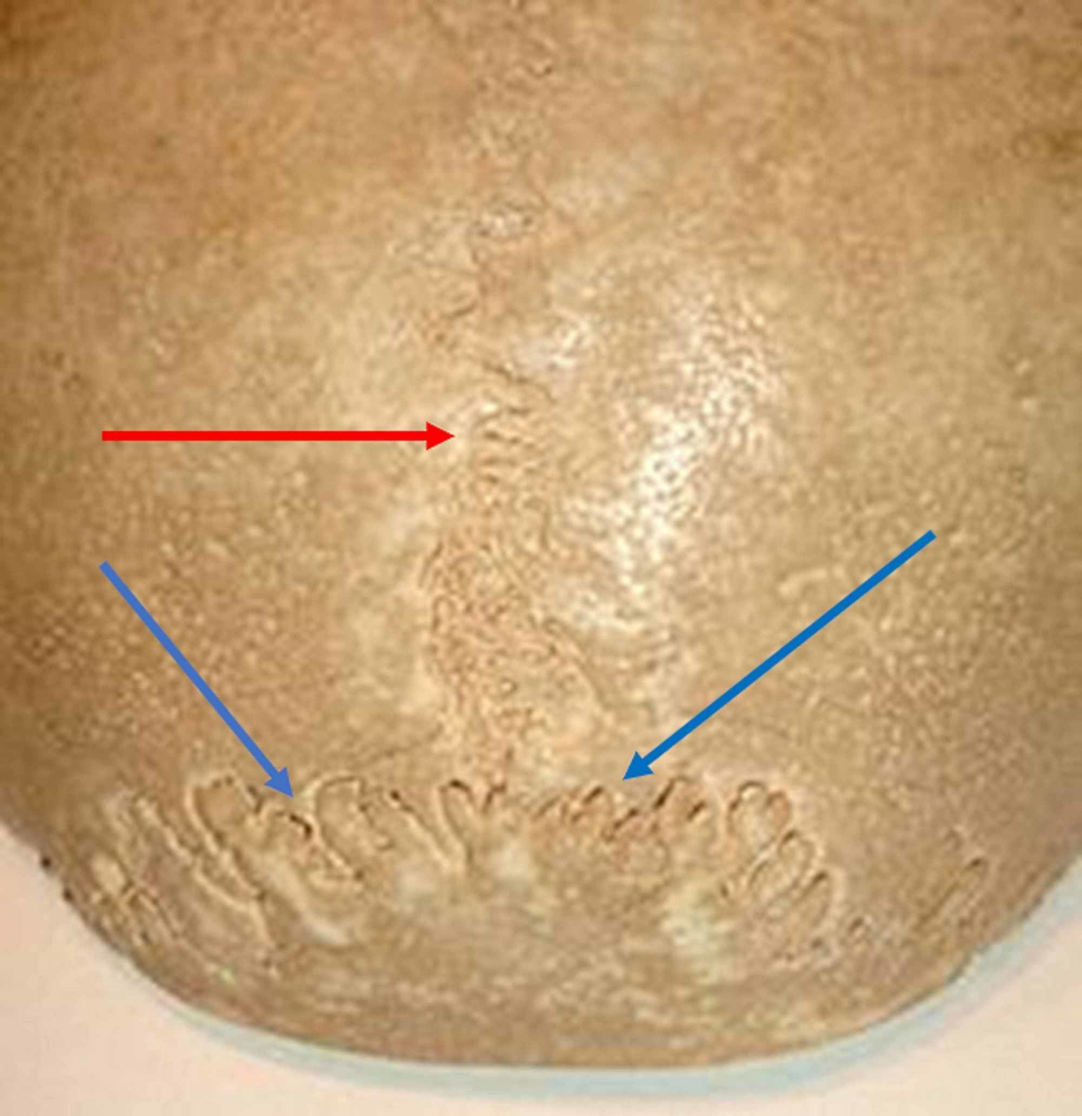
The figure illustrates the occipitoparietal sutures. The red arrow shows the suture between the parietal bones; the blue arrows show the occipitoparietal sutures.

The sutures consist of extracellular matrix, proteoglycans, collagen fibres and water; the synarthrosis with interdigitations, for example, the occipitoparietal, have a modulus of elasticity and absorption of mechanical stresses are greater (Figure 2) [4].

**Figure 3:**
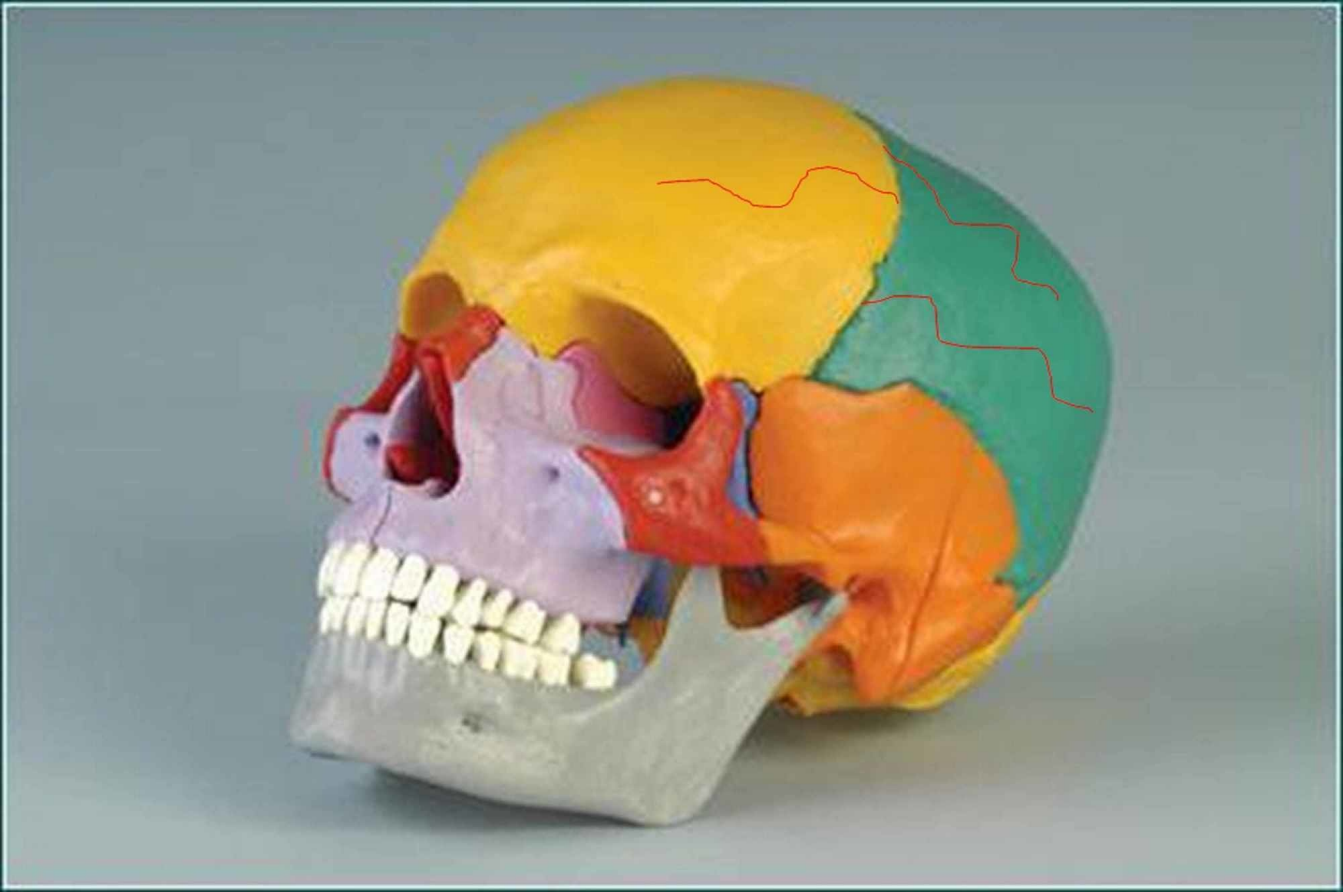
The figure shows a model of the skull with red lines drawn above the frontal bone and the parietal bone. The figure demonstrates the presence of trigeminal dural nerve endings external to the skull, whose terminations cross the sutures and innervate the myofascial system of the skull.

The sutures play different mechanical roles, including cushioning the extracranial tensions towards the skull and those intracranial towards the outside, thanks to the presence of cranial meninges [4]. We know that the cerebral mass moves (caudomedially and craniolaterally), through the solicitation of the heartbeat and the respiratory diaphragm [13]. The same cerebral mass, in particular, the neocortex and the limbic area oscillate during inspiration. The dura mater can change the mechanical tension of the extracranial musculature and the cervical tract, just as the deep pericranial musculature influences the mechanical dural tension. As regards the bi-univocal relationship of the dura mater and the pericranial musculature/fascia, the presence of trigeminal dural nerve endings external to the skull has been demonstrated; the latter cross the sutures and innervate the myofascial system of the skull (muscles and tendons) (Figure 3) [14-15].

To give an example of the myofascial presence above the bone tissue of the skull, the occipitofrontalis muscle has a very large tendinous arch below the galea capitis, which connects the muscular occipital body with the frontal one [16]. This muscle and other contractile and connective tissue districts that superficially cover the bones of the skull are involved by the dural afferents [17]. We cannot exclude the presence of sympathetic and parasympathetic terminations running parallel to the dural endings, with influence also on the intra/extracranial vascular pathways [17]. The dural afferents, beyond the extracranial area, reach the arachnoid space and the pia mater. These afferents/efferences function in an orthodromic and antidromic manner: there is a close relationship between the cranial myofascial system and the dura mater [17-19]. The relationship of the subtentorial dural area with the first cervical roots is always bi-univocal; the mechanical tension of the cervical musculature can be altered by dural information and the tentorial dural area can undergo a tension modification by the cervical musculature [20]. This happens due to afferents coming from the cervicovascular area and from non-trigeminal tentorial neural afferents reaching the cervical muscular portion [20]. The meningeal system, in which we recognize the dura mater, the arachnoid mater and the pia mater, is connective tissue; its fibroblasts can alter their tensional state, increasing or decreasing it [21]. By altering the dural tensional state, the secretion of some substances by fibroblasts (prostaglandins) is stimulated; from the sympathetic, parasympathetic and myelinated fibres terminations, multiple neuropeptides (noradrenaline, acetylcholine) are produced, as well as from the trigeminal meningeal terminations (calcitonin gene-related peptide - CGRP) [22-23]. These substances, depending on the perceived physiological or non-physiological stress, will influence the metabolic state of the brain and the brain's immune status, the repair capacity of the neuronal cells, as well as the nociceptive or analgesic meningeal response [22-23]. Recall that the meninges are rich in immune cells; the pia mater, with its outermost layer (glial limitans) and near the brain parenchyma, comes into contact with neuronal cells and astrocytes [23]. Palpation can be trained up to perceive measurable objects in microns and considering the close relationship of the cranial myofascial system and the intracranial meningeal system, we can throw new scientific reflections on the validity of osteopathic manipulative medicine (OMM). The tension of the hands resting on the skull/myofascial system is perceived by the extracranial trigeminal afferents, for example by improving brain oxygenation [24]. It must be remembered that there is a close relationship between the dural tissue and the blood vessels; an intra/extracranial mechanical stimulus easily alters dural vascular behaviour (permeability, vasodilation, vasoconstriction) [23]. The dura mater is vascularized and with the presence of lymphatic vessels; blood vessels pass molecules of low molecular weight or kilodalton (up to a maximum of 45kDa), which reach the arachnoid mater. The passage of larger molecules will depend on trigeminal intervention (vasodilation). The arachnoid mater, through the tight junctions, transports the blood molecules into the vessels of the subarachnoid space (in space we find different immune substances) [23]. The vessels of the subarachnoid space will enter the brain with the protection of the pia mater. The blood vessels and their perivascular connective tissue are considered a continuation of the pia mater [23]. We can hypothesize that the stimulation of extracranial trigeminal afferents could improve blood transport, from dura to pia, improving arterial vasodilation. By improving extracranial tension, it improves the intracranial trigeminal response [25]. It must be considered that at the cellular level of the different cranial tissues there are no layers, but an absolute anatomical and functional continuity [26-27]. Quantum physics helps us further. Palpation is interactive communication between the operator and the patient, and all the palpated and not palpated tissues are aware of the mechanical information that comes from the hands placed on the skull (quantum entanglement) [27]. Magnetobiology relates electromagnetic fields and living cells [27]. The operator's hand emits electromagnetic fields (such as on the patient's skull), and these magnetic forces or vibrations deform the morphology of the cell, becoming a mechanical stimulus felt by extracranial terminations trigeminal. The electromagnetic fields travel at higher speeds than the electric flow, crossing the whole body; the touch of the osteopath goes beyond the skull [27]. The fact that the cranial sutures are still patent in old age, could mean that the millimetre movement of the brain during systole/diastole and the contractions of the diaphragm muscle, is amortized by cranial synarthrosis. Probably, the oscillations of some brain areas, such as the hippocampus and the limbic area during breathing, could influence the same movement of the brain mass. Does OMM affect the liquids in the skull? All cells oscillate and aggregate to form tissues; liquids are an important fascial component and form the liquid fascia [28]. Thus we have the extracellular matrix, the interstitial fluids, blood, lymph, liquor and the same cells that are full of water [27]. The oscillations of the cells create further alterations of mechanical tension that travel faster in the liquid tissues, creating a wet network [27]. It is very probable, relying on the notions of physics that the OMM is able to enter into communication with the liquor or the cranial lymph. Can we feel the movement of the cranial bones? The overall movement of the cranial bones, allowed by the patency of the various sutures, is measured in microns, with an amplitude that is around 10-50 μm [29]. The palpatory sensitivity of the operator trained to listen to the smallest movements and variations of tension coincides with the measure of cranial movement [29]. We still need a lot of information on what happens between the osteopath, his hands and the patient's skull, but it is this need to know which creates the research, the right orientation towards knowledge. The question must always be neutral in order to obtain an efficient scientific response and move forward: this is the enlightenment of research.

## Conclusions

The cranial osteopathic manipulative medicine is not always positively shared by the scientific world, but it cannot even be rejected by scholars and scientists since non-knowledge does not preclude the end of knowledge. The article discussed an orientation of the current literature that goes against the scientific nature of the osteopathic manipulative medicine (OMM), showing, however, that there are further reflections that are able to be made in support of osteopathic cranial therapy.References
